# Executive function performance in middle-aged adults

**DOI:** 10.1590/1980-5764-DN-2022-0065

**Published:** 2023-05-29

**Authors:** Namrata Sharma, Shweta Shenoy

**Affiliations:** 1 Guru Nanak Dev University, MYAS-GNDU Department of Sports Sciences and Medicine, Amritsar (Punjab), India.

**Keywords:** Middle Aged, Executive Function, Neuropsychological Tests, Education, Gender Role, Pessoa de Meia-Idade, Função Executiva, Testes Neuropsicológicos, Educação, Papel de Gênero

## Abstract

Executive functions have been widely studied in the extreme of ages, but studies in middle-aged adults remain largely neglected. Education and gender are known to influence cognitive performance; however, their effect on executive function in middle-aged adults remains unclear.

**Objective:**

The study aimed to observe the effect of hierarchy of educational qualifications (graduate, postgraduate, and PhD) and gender on various executive function tests across middle-aged adults with or without comorbidity.

**Methods:**

A total of 66 middle-aged individuals volunteered for the study (mean age=48.45±5.45 years; 20 graduates, 28 postgraduates, and 18 PhD; 36 males and 30 females; 38 healthy adults and 28 adults with comorbidities). Each subject performed a test assessing short-term memory, spatial working memory, and multitasking abilities on the Cambridge Neuropsychological Test Automated Battery with rest periods in no specific order of tests. Key parameters of cognitive tests were analyzed for differences in educational qualifications (ANOVA), gender (t-test), and the effect of comorbidity as a covariate (ANCOVA).

**Results:**

PhDs performed significantly better (p<0.05) in multitasking than graduates and had superior visuospatial working memory (fewer errors). Differences in simultaneous matching abilities, lower incongruence cost and multitasking cost were statistically significant in healthy females than in males.

**Conclusion:**

On considering adults with comorbidity, those with higher educational attainment retained the ability to multitask compared to their healthy counterparts, which was not seen in the group with lower educational attainment. Thus, higher educational attainment attenuated the influence of comorbidities and deterioration of executive functions in general in middle-aged adults.

## RESUMO.

As funções executivas têm sido amplamente estudadas no extremo das idades, mas os estudos na meia-idade permanecem amplamente negligenciados. Educação e gênero são conhecidos por influenciar o desempenho cognitivo, no entanto, seu efeito na função executiva em adultos de meia-idade ainda não está claro.

**Objetivo:**

O estudo teve como objetivo observar o efeito da hierarquia de qualificações educacionais (graduação, pós-graduação e doutorado) e gênero em vários testes de função executiva em adultos de meia-idade com ou sem comorbidade.

**Métodos:**

Sessenta e seis indivíduos de meia idade se voluntariaram para participar do estudo (idade média=48,45±5,45 anos, 20 graduados, 28 pós-graduados e 18 doutores; 36 homens e 30 mulheres; 38 saudáveis e 28 adultos com comorbidades). Cada sujeito realizou um teste avaliando memória de curto prazo, memória de trabalho espacial e habilidades multitarefa na Bateria Automatizada de Testes Neuropsicológicos de Cambridge (*Cambridge Neuropsychological Test Automated Battery* — CANTAB) com períodos de descanso em nenhuma ordem específica de testes. Os principais parâmetros dos testes cognitivos foram analisados quanto às diferenças nas qualificações educacionais (ANOVA), sexo (teste *t*) e efeito da comorbidade como uma covariável (ANCOVA).

**Resultados:**

Os doutores tiveram desempenho significativamente melhor (p<0,05) em multitarefa do que os graduados e tiveram memória de trabalho visual-espacial superior (menos erros). As diferenças nas habilidades de correspondência simultânea, menor custo de incongruência e custo de multitarefa foram estatisticamente significativas em mulheres saudáveis do que em homens.

**Conclusão:**

Ao considerar os adultos com comorbidade, aqueles com maior escolaridade mantiveram a capacidade de multitarefa como seus pares saudáveis, o que não foi observado no grupo com menor escolaridade. Assim, maior escolaridade atenuou a influência de comorbidades e deterioração das funções executivas em geral, em adultos de meia-idade.

## INTRODUCTION

Executive function (EF) is a hypernym for the complex cognitive processes that underlie flexible, goal-directed behavioral responses to novel or difficult situations. There is a general consensus that there are three core EFs or subdomains, namely, inhibition, working memory (WM), and cognitive flexibility^1^. These higher order EFs contribute to reasoning, problem-solving, and planning^2,3^. Short-term memory also plays a significant role in contributing to executive functioning^4^.

The majority of studies have examined binary young/old adult age groups, and just a few studies have looked into age-related changes in EF in middle-aged adults or adolescents^5^. Ferguson et al.^6^ suggested that middle-aged population is largely overlooked and highlighted the need of including them to provide a comprehensive life span description of the distinct developmental trajectories of EFs.

Previous studies have suggested that education influences cognitive functioning. Lower educational attainment is associated with poorer cognitive functioning in middle-aged^7^ and older adults^8,9^. However, findings regarding cognitive change are mixed^10^. The role of educational attainment as a factor in contributing to the cognitive performance specific to middle-aged adults remains elusive.

Gender variations in connection to cognitive aging have also been investigated. Hughes et al.^7^ suggested that in middle-aged adults, women outperform men on episodic memory tasks including immediate and delayed word recall. Middle-aged men outperform women on specific memory tests, particularly those with an analytic or spatial component^11,12^. The subject of whether men and women have different change trajectories throughout midlife and beyond is still a topic of debate.

The present research was planned in an attempt to gain insight into the cognitive performance of middle-aged adults, mainly EF. We employed the Cambridge Neuropsychological Test Automated Battery (CANTAB) computerized test batteries to assess EF performance^13–15^ in our research. The first objective of our study was to observe the effect of educational qualification and gender on various tests of CANTAB challenging the short-term memory (DMS), visuospatial working memory (VSWM), and multitasking (MTT) in middle-aged adults, that is, between the ages of 40 and 60 years, according to the American Psychological Association^16,17^. The participants were classified on the basis of their educational qualifications, namely, graduate (G), postgraduate (PG), and doctorate of philosophy (PhD) groups. For the study to be representative of a true cross section of the middle-aged population, we did not exclude subjects on account of any comorbid condition they may have. Instead, we chose to analyze and compare the comorbid group with individuals without comorbidity, considering it as our second objective. Based on the existing literature, it was hypothesized that a higher educational qualification that is PhD will contribute to better accuracy in cognitive scores DMS, SWM, and MTT in comparison to a lower educational qualification that is G; however, reaction latencies may not be influenced. Second, gender differences would be evident in cognitive scores with females having better scores on DMS in comparison to males since females have better short-term memory^7^. Third, males will have better scores (fewer errors) in SWM task^18,19^. Fourth, individuals with comorbidities will perform worse than healthy individuals on EF tasks^20^.

## METHODS

Middle-aged participants working/living on the university campus were asked to volunteer for the study via advertisements put up on notice boards in the administrative and academic blocks. The inclusion criteria of the study were as follows:

- Participants within the age range of 40–60 years;
- Participants who had completed formal education up to class 12; and
- Participants who were able to understand English language.

Participants who scored above 26 in the Mini-Mental State Examination^21^, a scale for intact cognitive function, met the inclusion criteria for the study. In all, 66 middle-aged adults employed at the Guru Nanak Dev University in various teaching and non-teaching positions were recruited. The following information was gathered on their educational qualification.

- Those who had completed any undergraduate course after 3 years of study were regarded as graduates (G=20).
- A postgraduate was someone who had completed a 2-year degree, following an undergraduate course (PG=28).
- A PhD was someone who has completed doctoral level coursework (PhD=18).

Self-reported sleeping hours for the night before testing were documented. The experiment was approved by the university ethics committee and complied with the ethical standards as laid down in the Declaration of Helsinki. Participants read and signed an informed consent statement before the beginning of the experimental session.

### Cambridge Neuropsychological Test Automated Battery (CANTAB)

All participants were seated at a comfortable height and handed the CANTAB I-pad^22^ to carry out the EF test by placing responses on a touch screen. Each test began with practice items at a basic level.

#### Delayed matching of sample

This level assessed both simultaneous and short-term visual memory. Participants were shown a figure composed of different colors and abstract patterns. In a four-choice recognition test of abstract patterns that share color or pattern with distracters, they were asked to choose the correct response simultaneously or after the figure got covered up and options appeared either immediately or after 4 or 12 s.

#### Spatial working memory

This is a self-ordered search test assessing nonverbal WM. Participants were asked to search through several colored boxes presented on the screen to find yellow tokens hidden inside. Each box contains only one token per trial. With each stage, the number of colored boxes kept on increasing.

#### Multitasking

This test measures the participant's ability to use multiple sources of potentially conflicting information to guide behavior and to ignore task-irrelevant information, posing a Stroop-like effect. During the test, an arrow appeared on either side of the screen or pointed in both directions. Before each trial, a cue was displayed at the top of the screen to indicate to the participant whether they must push the right or the left button, considering the cue to identify the side or the direction of the arrow. The initial part of the tests included sections during which the rule is consistent across trials that are either side of arrow or direction of the arrow (single task), and during the latter part of the sections of the test, the rule may randomly change from trial to trial (MTT).

#### Procedure

The demographic information of each participant was gathered. Self-reported presence of comorbidity was noted. In all, 6 people had diabetes, 8 participants had hypothyroidism, and 13 participants had hypertension. Each EF task began with a familiarization session for all participants. They were given vocal instructions with visual graphics, and then each activity had a demonstration built in before it was started. To prevent any bias in test results, the cognitive task's order was kept random (Table 1).

**Table 1:** Demographics of the sample population.

Characteristics	n=66
Level of education	Graduate: 20 (M=11 and F=9; mean age=49.3±4.5; healthy subjects=12 and with comorbidity=8)
	Postgraduate: 28 (M=21 and F=7; mean age=47.7±6.1; healthy subjects=17 and with comorbidity=11)
	PhD: 18 (M=4; and F=14; mean age=48.5±5.2; healthy subjects=9 and with comorbidity=9)
Gender	Males: 36 (mean age=49.1±5.9; G=11; PG=21; PhD=4)
	Females: 31 (mean age=47.5±4.7; G=9; PG=7; PhD=14)
Job profiles	Administrative (n=1)
	Business (n=4)
	Clerical (n=17)
	Executive (n=17)
	Housewife (n=5)
	Teaching (n=23)
Health status	Healthy adults: 38 (G=12; mean age=47.5±3.47; PG=17; mean age=45.8±5.4; PhD=9; mean age=47.4±5.54)
	Adults with comorbidity: 28 (G=8; mean age=51.7±5.14; PG=11; mean age=50.4±6.41; PhD=9; mean age=49.7±5.17) Types of comorbidities: diabetes (6), hypertension (13), hypothyroidism (8)

Abbreviations: F: female; G: graduate; M: male; PG: postgraduate; PhD: doctorate of philosophy.

### Statistical analysis

A priori power analysis was conducted before the study (G*power), which estimated that the minimal sample size needed was 66 participants (effect size=0.4, err prob=0.05, power 1-β err prob=0.8). A total of 68 subjects were recruited for the study; however, two subjects did not complete all the required tests, and we completed the study with 66 subjects. Statistical analysis was done using the IBM SPSS version 23 (IBM, USA) software. The statistical significance level was set at p<0.05. Since our sample population consisted of 28 comorbid individuals out of 66 individuals, analysis was done in the whole group (healthy=38, with comorbidity=28) and separately on healthy individuals. A one-way analysis of variance (ANOVA) test was applied based on their educational qualifications (G=20, PG=28, PhD=18) to the whole group and subgroups of healthy individuals and comorbid individuals. Both group-wise comparisons were made between key outcome measures of DMS, SWM, and MTT according to gender, as well as gender differences in the healthy population (males=24; females=14) that were analyzed using an independent t-test. Since approximately 41% of the participants were having some comorbidities, we decided to examine the presence of comorbidity as a covariate. An analysis of covariance (ANCOVA) was also applied to keep the presence of comorbidity as a covariate, across subgroups of educational qualifications.

## RESULTS

A total of 66 middle-aged individuals (mean age=48.45±5.45 years, 20 G, 28 PG, and 18 PhD) were included in the study.

### Changes in executive function scores according to education qualification

ANOVA was applied on the basis of level of education. Since values of reaction latencies and MTT correct scores were found to be heterogeneous and unequal variances were found; Welch p-values and Brown-Forsythe values were considered instead of F-values. On analyzing MTT parameters, MTT correct scores were significantly higher at F=7.73, df (2, 65), and p<0.001 in PhDs in comparison to G (Table 2).

**Table 2:** ANOVA test on whole group (G=20, PG=28, PhD=18).

Cognitive scores		G	PG	PhD	Homogeneity p-value	F value	p-value	Welsch test p-value	Brown-Forsythe p-value
DMS	DMSTC	16.7±1.5	17.2±1.8	17.1±2	0.749	0.435	0.64	0.61	0.65
	DMSTCAD	11.8±1.5	12.3±1.7	12.1±1.9	0.63	0.43	0.65	0.62	0.65
	DMSML	3491.69±851.6	3803.96±1059.5	3281.14±627.4	0.034	1.95	0.15	0.12	0.12
	DMSMLAD	3559.36±818.9	4000.16±1230.2	3375.9±676.7	0.017	2.46	0.093	0.10	0.064
	DMSMLS	3289.14±1114.6	3349.67±991.9	3049.33±785.27	0.67	0.53	0.58	0.51	0.58
	DMSPEGC	0.17±0.08	0.14±0.10	0.14±0.10	0.77	0.85	0.43	0.39	0.42
SWM	SWMTE	17±8.3	15±10.0	10.9±6.8	0.18	2.35	0.10	0.05	0.08
	SWMBE	15.8±8.0	13.8±8.9	10.1±6.6	0.22	2.43	0.09	0.05	0.08
	SWMBE12	36.25±7.9	37.42±12.3	33±14.6	0.03	0.76	0.46	0.57	0.47
	SWMSX	16.4±2.7	16.5±2.4	15.9±3.2	0.77	0.25	0.77	0.81	0.79
MTT	MTTC	130±24.8	148.7±18.4	152.2±12.2	0.001	7.73	0.001	0.004*	0.001
	MTTML	907.1±121.2	876.88±89.4	891.77±106.1	0.40	0.49	0.61	0.63	0.63
	MTTMLMT	1023.94±175.9	1001.16±90.9	1014.72±125.72	0.003	0.18	0.83	0.83	0.84
	MTTLNOM	924.15±133	907.00±91.1	909.9±112.6	0.41	0.14	0.86	0.88	0.87
	MTTICOST	33.8±68.7	60.1±61.1	36±84.2	0.97	1.05	0.35	0.33	0.38
	MTTMTCM	230.75±139.4	246.35±117.5	243.35±126.4	0.52	0.09	0.91	0.92	0.91

Abbreviations: G: graduate; PG: postgraduate; PhD: doctorate of philosophy; DMS: delayed matching to sample; DMSTC: DMS total correct; DMSTCAD: DMS total correct all delay; DMSML: DMS reaction latency; DMSMLAD: DMS reaction latency to all delay; DMSMLS: DMS reaction latency to simultaneous task; DMSPEGC: DMS probability of error given error; SWM: spatial working memory; SWMTE: SWM total errors; SWMBE: SWM between errors; SWMBE12: SWM between errors when 12 tokens are present; SWMSX: SWM strategy in 6–12 boxes; MTT: multitasking; MTTC: MTT correct score; MTTML: MTT reaction latency; MTTMLMT: MTT reaction latency during multitask condition; MTTLNOM: MTT reaction latency during incongruent condition; MTTICOST: MTT incongruence cost; MTTMTCM: MTT multitasking cost.

* Notes: p-value of MTTC was significant at 0.004.

**Figure 1:**
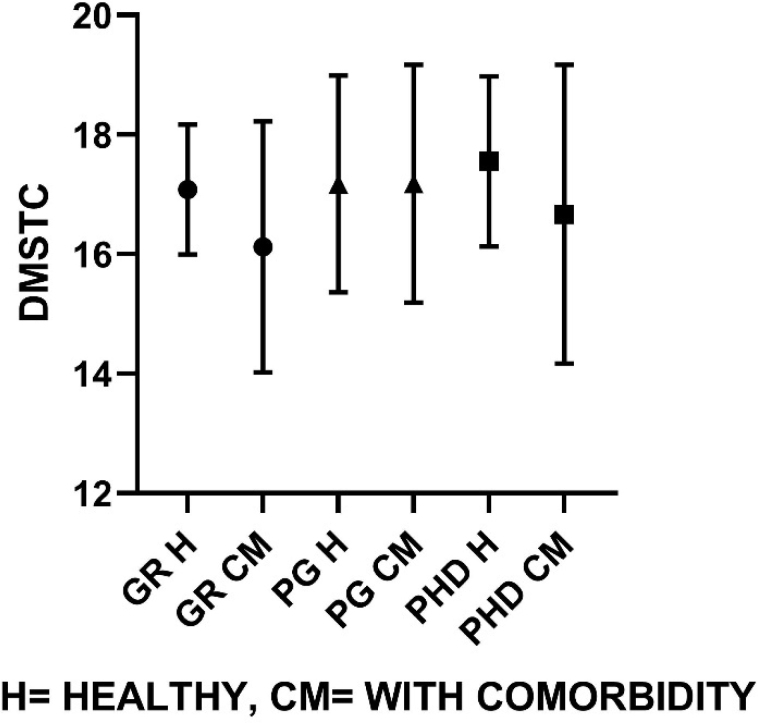
Delayed matching to sample total correct mean values for healthy population and comorbid population, respectively (G=17.08±1.1, 16.1±2.1; PG=17.17±1.8, 17.1±1.9; PhD=17.55±1.4, 16.6±2.5).

Abbreviations: G: graduate; PG: postgraduate; PhD: doctorate of philosophy.

**Figure 2:**
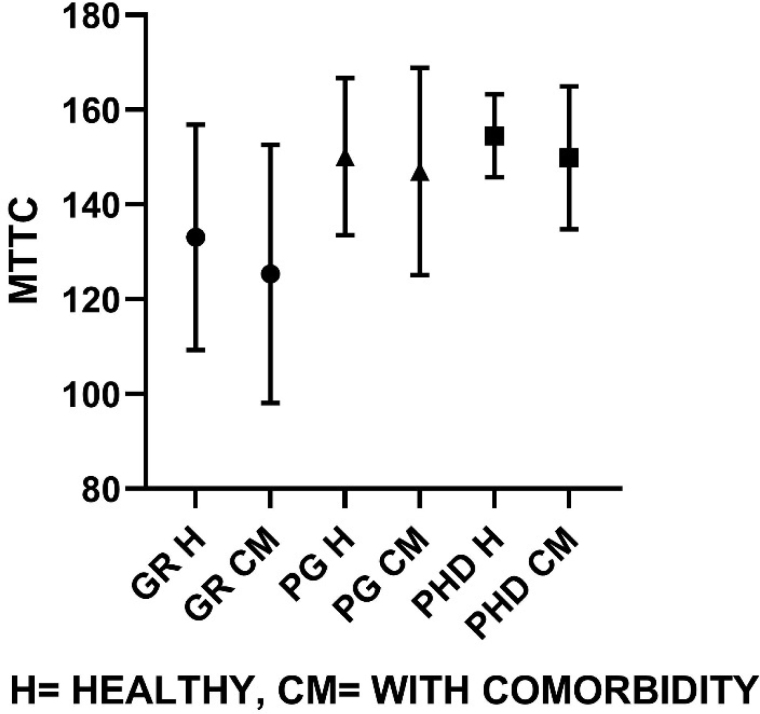
Multitasking mean correct scores for healthy population and comorbid population, respectively (G=133.08±23.8, 125.3±27.2; PG=150.11±16.5, 147±21.9; PhD=154.55±8.7, 149.8±15.1).

Abbreviations: G: graduate; PG: postgraduate; PhD: doctorate of philosophy.

**Figure 3:**
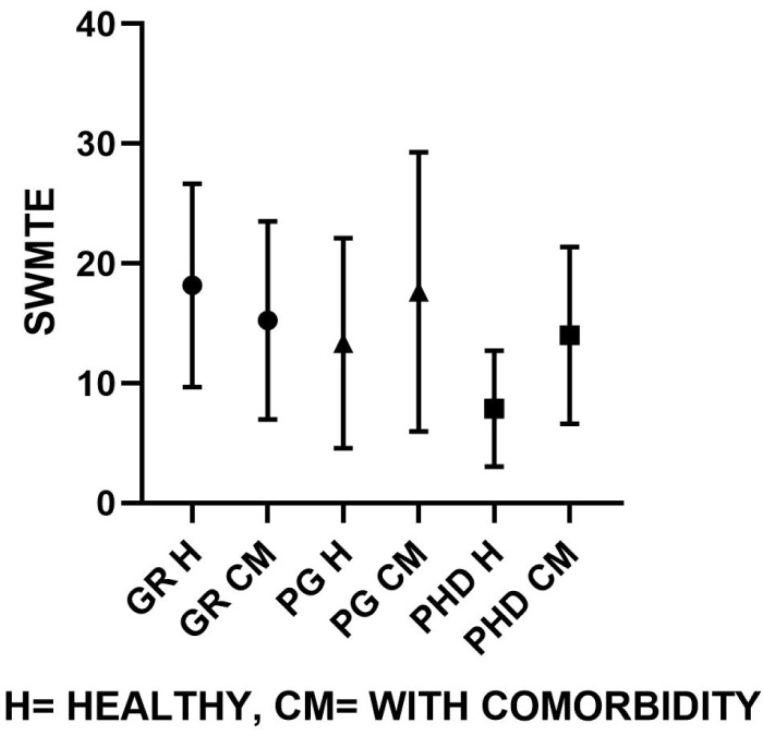
Spatial working memory mean total errors for healthy population and comorbid population, respectively (G=18.2±8.4, 15.2±8.2; PG=13.3±8.7, 17.6±11.6; PhD=7.8±4.8, 14±7.3).

Abbreviations: G: graduate; PG: postgraduate; PhD: doctorate of philosophy.

**Figure 4:**
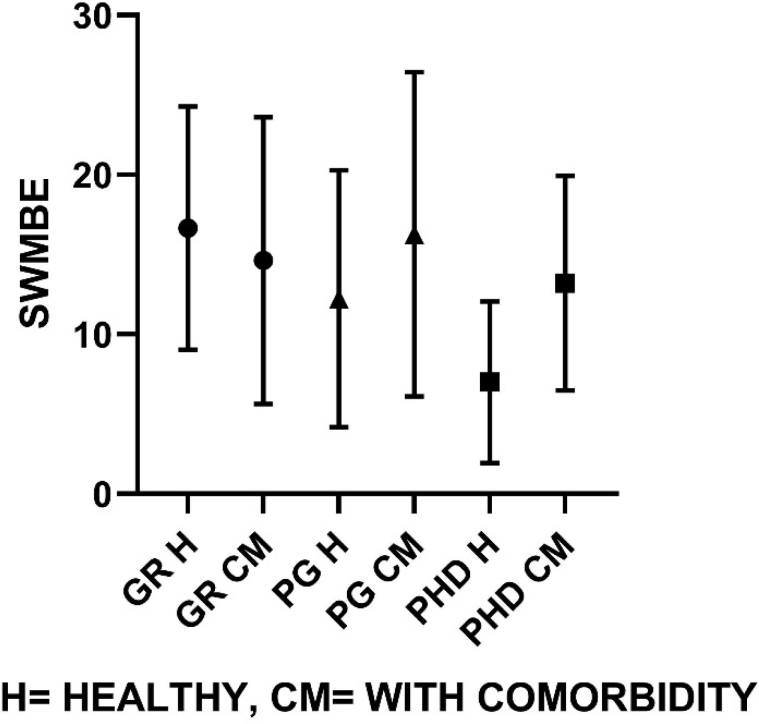
Spatial working memory mean between errors for healthy population and comorbid population, respectively (G=16.6±7.6, 14.6±8.9; PG=12.2±8.1, 16.2±10.1; PhD=7±5.07, 13.2±6.7).

Abbreviations: G: graduate; PG: postgraduate; PhD: doctorate of philosophy.

Similarly, group-wise comparisons according to their educational classification were made on groups of healthy individuals and comorbid individuals represented graphically (Figures 1–4). On comparison of means in the healthy group classified on the basis of educational level, the SWM total errors and between errors had a statistical significance at F=4.31, df (2, 37), p<0.05, and F=4.48, df (2, 37), p<0.05 levels, respectively, where both these variables were gradually turned down with the increase in educational qualification. Among all MTT variables, the correct score only showed significant changes with higher educational qualification, F=4.58, df (2, 37), p<0.05. The post hoc test revealed that PhD and PG groups have significantly higher MTT correct values than graduate degree holders (p<0.05).

### Changes in executive function scores according to gender

On analyzing the whole group, since unequal samples were compared between males (n=36) and females (n=30), p-value with assumed unequal variances was taken into consideration. Results of the independent t-test inferred that DMS total correct scores were marginally higher in females than males. Reaction latency in a simultaneous task (overt condition) was statistically significantly lower in females (t=2.4; p<0.05) compared to males (Table 3 and 4).

**Table 3:** T-test on whole group (males=36, females=30).

Cognitive scores		Males	Females	t-value	p-value
DMS	DMSTC	16.9±1.7	17.03±1.8	−0.21	0.83
	DMSTCAD	12.1±1.6	12.1±1.8	0.013	0.99
	DMSML	3686.14±991.4	3423.48±796.7	1.17	0.24
	DMSMLAD	3762.4±1086.9	3617.04±919.2	0.58	0.55
	DMSMLS	3496.76±1060.47	2952.61±776.4	2.40	0.019*
	DMSPEGC	0.15±0.09	0.15±0.11	0.23	0.82
SWM	SWMTE	14.63±8.2	14.36±9.7	0.12	0.90
	SWMBE	13.6±7.9	13.2±8.8	0.19	0.84
	SWMBE12	34.5±12.9	37.46±10.4	−1.01	0.31
	SWMSX	16.1±2.7	16.6±2.7	−0.71	0.48
MTT	MTTC	143.72±20.8	144.3±22	−0.12	0.91
	MTTML	880.33±108.87	901.82±97.42	−0.83	0.39
	MTTMLMT	1004.29±135.87	1020.72±122.64	−0.51	0.61
	MTTLNOM	909.09±116.76	917.67±101.98	−0.31	0.75
	MTTICOST	57.2±62.2	31.6±77.7	1.45	0.15
	MTTMTCM	245.45±132.1	235.36±118.1	0.32	0.74

Abbreviations: DMS: delayed matching to sample; DMSTC: DMS total correct; DMSTCAD: DMS total correct all delay; DMSML: DMS reaction latency; DMSMLAD: DMS reaction latency to all delay; DMSMLS: DMS reaction latency to simultaneous task; DMSPEGC: DMS probability of error given error; SWM: spatial working memory; SWMTE: SWM total errors; SWMBE: SWM between errors; SWMBE12: SWM between errors when 12 tokens are present; SWMSX: SWM strategy in 6–12 boxes; MTT: multitasking; MTTC: MTT correct score; MTTML: MTT reaction latency; MTTMLMT: MTT reaction latency during multitask condition; MTTLNOM: MTT reaction latency during incongruent condition; MTTICOST: MTT incongruence cost; MTTMTCM: multitasking cost.

* Notes: p-value of DMSMLS was significantly at 0.019.

**Table 4:** T-test on healthy group (males=24, females=14).

Cognitive scores		Male	Female	t-value	p-value
DMS	DMSTC	17.0±1.5	17.5±1.3	−1.1	0.28
	DMSTCAD	12.2±1.5	12.6±1.2	−1.03	0.31
	DMSML	3589.19±879.8	3526.3±781.2	0.22	0.82
	DMSMLAD	3726.86±1084.2	3755.66±889.2	−0.08	0.93
	DMSMLS	3276.82±746.5	2963.25±737.1	1.25	0.21
	DMSPEGC	0.15±0.09	0.12±0.06	1.41	0.16
SWM	SWMTE	13.8±8.3	13.1±9.4	0.22	0.82
	SWMBE	12.7±7.7	11.8±8.6	0.30	0.76
	SWMBE12	33±15.1	37.2±11.2	−0.99	0.32
	SWMSX	16.1±2.9	16±2	0.21	0.83
MTT	MTTC	145.08±20.1	147±19.2	−0.29	0.77
	MTTML	860.5±105.6	893.7±112.5	−0.89	0.37
	MTTMLMT	1001.12±147.06	991.23±132.4	0.213	0.83
	MTTLNOM	895.6±121.8	907.7±116.3	−0.31	0.76
	MTTICOST	69.8±61.3	28.1±45.8	2.38	0.023*
	MTTMTCM	278.26±121.9	194.61±111.3	2.15	0.039*

Abbreviations: DMS: delayed matching to sample; DMSTC: DMS total correct; DMSTCAD: DMS total correct all delay; DMSML: DMS reaction latency; DMSMLAD: reaction latency to all delay; DMSMLS: reaction latency to simultaneous task; DMSPEGC: DMS probability of error given error; SWM: spatial working memory; SWMTE: SWM total errors; SWMBE: SWM between errors; SWMBE12: SWM between errors when 12 tokens are present; SWMSX: SWM strategy in 6–12 boxes; MTT: multitasking; MTTC: MTT correct score; MTTML: MTT reaction latency; MTTMLMT: MTT reaction latency during multitask condition; MTTLNOM: MTT reaction latency during incongruent condition; MTTICOST: MTT incongruence cost; MTTMTCM: MTT multitasking cost.

* Notes: p-values of MTTICOST and MTTMTCM were significant at 0.023 and 0.039, respectively.

### Effect of level of education on executive function scores keeping presence of co morbidity as a covariate

ANCOVA was applied to see the effect of level of education on EF scores with the presence of comorbidity as a covariate. MTT correct score was found to be significantly higher in adults with a PhD degree despite the presence of comorbidity in comparison to adults with graduate degrees and comorbidity, at F=5.58, df (3, 62), p<0.05.

## DISCUSSION

Our study aimed to explore the effect of gender and educational qualification on EF scores of middle-aged adults with or without comorbidity. Our hypothesis that males and females would fair differently in various EF tasks challenging various subdomains of EF was proved. Another hypothesis that adults with higher educational qualification would perform better in EF tests than those with lower educational qualification was confirmed through our results similar to previous literature^10,23^. Since individuals with comorbidity were also included in the sample population, EF scores were compared in all samples (healthy subjects+subjects with comorbidity) and the subpopulation of healthy samples for gender differences and the influence of education. It is interesting to note that in our study, the PhD group had an equal number of healthy individuals with comorbidity in comparison to graduate and postgraduate groups, which had more healthy individuals than individuals with comorbidity, yet the PhD group demonstrated higher EF scores in comparison to their counterparts. Thus, it appears that an increase in years of education may have led to protection against the negative consequences of aging on cognition.

In the DMS task, those with higher educational qualification and females performed distinctively. Trends indicated faster processing speed and retrieval, in both visual matching abilities and visual short-term memories of information in the PhD group in comparison to the other groups. Salthouse^24^ found correct scores were higher in females in comparison to males; on the contrary, reaction latencies, which indicate the speed of processing, were also lower in females in comparison to males. Voyer et al.^25^ found evidence favoring object identity memory greater in females over males. Statistically significant differences were only seen in reaction latency of simultaneous tasks, which is also referred to as the overt condition. According to Mammarella et al.^26^, DMS overt condition is regarded as a better indicator of simultaneous matching abilities. Dahlberg^27^ and Jordan^28^ suggested that increased simultaneous matching abilities in the case of females may be accredited to human evolution, where according to hunter-gatherer gender roles, men used to hunt animals for protection and food and women used to gather the plant food that often accounted for the meal. Hence in women, a superior pattern matching ability would be of an evolutionary advantage to recognize edible, poisonous foliage, and fruit. Reaction latencies in delayed tasks indicated better retrieval of information from memory in females, which has been demonstrated in a previous study^29^.

Gender differences in SWM were found to be greater in PhDs than the others and modest among gender. The performance of the task involved that the subject had to search for tokens in spatially arranged boxes, with a single token appearing in each box once and the participant had to remember the box where the token was previously found to avoid between errors. The number of boxes increased sequentially with 4, 6, 8, and 12 boxes by the end of the task. The task challenged the VSWM in the initial stages of the task; however, as the workload increased with 8 and 12 boxes in the subsequent stages, the participants had to develop a cognitive strategy to complete the task^30^. Zarantonello et al.^19^ concluded that educational level had a positive effect on WM performance on both reaction times and accuracy. Our study supports this literature with the PhD group demonstrating a lower number of total errors at all levels of test difficulty including the 12 token stages which involved the application of strategy as well as VSWM for performance execution.

On comparing scores between genders in the SWM task, the mean values of total errors and between errors were almost similar, which may reflect the near-ceiling effect of the test reported previously as well^31^. However, as the task complexity increased (at the 12-token stage), the number of errors was slightly lower in males in comparison to females (in the healthy+with comorbidity group and healthy group). Since the SWM task not only focuses on VSWM but also involves strategy as a key component, it is unclear if better results in men are by virtue of superior VSWM or cognitive strategy. Differentiating among these key components could be areas of further research.

Multitasking abilities were also enhanced as years of education increased, with our results demonstrating that PhDs were more receptive to addressing multiple stimuli and suppressing irrelevant stimuli in comparison to those with lower education qualification. The processing speed in MTT (indicated by MTT cost and incongruence cost) was found to be even superior in healthy adults with a higher degree in comparison to healthy adults with a lower degree. According to Tun and Lachman^32^, higher education (a college degree) was linked with significantly faster response latencies. However, when observing the whole group in our study, incongruence cost and MTT cost were lowest in graduates, although this can be attributed to speed accuracy trade-offs, indicating overall poor performance by the former group in the task.

Females were at an advantage in comparison to males in the MTT task. But males were found to have an advantage in reaction latency in comparison to females, in coherence with previous results by Der and Deary^33^. Both incongruence cost and MTT cost were found to be lower in the sample of all females (healthy+with comorbidity) in comparison to all males (healthy+ with comorbidity). However, the trends were found to be statistically significant (p<0.05) when only healthy women (n=14) and healthy men (n=24) were compared, excluding individuals with comorbidity, indicating clearly that females have superior MTT and cognitive inhibition and that having comorbid conditions may have compromised these abilities.

The graduate and postgraduate groups had a higher number of healthy individuals than the PhD group, which had an equal number of healthy and comorbid individuals. It is interesting to note that the PhD group had superior scores in 12 out of the total 16 key parameters assessed by CANTAB. These results reinforce the theory of cognitive reserve. Cognitive reserve is the ability to optimize or maximize performance through differential recruitment of brain networks, which perhaps reflect the use of alternate cognitive strategies^34^. Individuals with higher degrees may have performed well since education seems to boost abstract thinking abilities, allowing individuals to tackle more complicated problems^35^ and achieve higher scores in EF tasks. In addition, such individuals have demanding occupational requirements requiring higher-order thinking on a daily basis.

The effect of level of education keeping the presence of comorbidity as a covariate was also investigated. A distinct finding here highlighted that MTT was significantly improved in individuals with higher educational qualification even in the presence of comorbidity in comparison to their counterparts with lower educational qualification. It is well established that cognitive performance is hampered in hypothyroidism^36–39^, hypertension^40^, and diabetes^41^, and cognitive decrement may start early in an individual with these comorbidities in comparison to controls. Educational attainment, a proxy measure for cognitive reserve, may be responsible for actively resisting the changes brought about by age and disease^42,43^. This may have ensured a better score in the EF task despite the presence of comorbidity in individuals with PhD in the present study. Our results may have broader implications for separate studies on each comorbid condition, i.e., hypothyroidism, diabetes, and hypertension to confirm the present findings of education offsetting the changes in cognition due to the aforementioned comorbidities.

Since our study was delimited to an educated working population, it was assumed that the participants met average IQ requirements. However, the fact that we did not actually measure their IQ could be considered a limitation of this study. Another limitation is the small sample size of the study.

Despite this limitation, our study provides clear evidence for greater cognitive reserve and perhaps plasticity in middle age across conditions that could potentially influence EF, with more years invested in education influencing task performances.

Among middle-aged adults, better educational attainment reduced the impact of comorbidities and the general decline in executive functions.
